# Sleep Inconsistency and Markers of Inflammation

**DOI:** 10.3389/fneur.2020.01042

**Published:** 2020-09-16

**Authors:** Joseph M. Dzierzewski, Emily K. Donovan, Daniel B. Kay, Timothy S. Sannes, Keighly E. Bradbrook

**Affiliations:** ^1^Department of Psychology, Virginia Commonwealth University, Richmond, VA, United States; ^2^Department of Psychology, Brigham Young University, Provo, UT, United States; ^3^Division of Psychosocial Oncology and Palliative Care, Dana-Farber Cancer Institute, Boston, MA, United States; ^4^Harvard Medical School, Boston, MA, United States; ^5^Department of Biostatistics, Virginia Commonwealth University, Richmond, VA, United States

**Keywords:** sleep, actigraphy, biomarkers, cytokines, multivariate analyses, psychoneuroimmunology

## Abstract

**Objective:** Poor sleep is associated with higher levels of inflammatory biomarkers. Conventionally, higher average time awake, lower average time asleep, and lower sleep efficiency define poor sleep. Recent research suggests that, in addition to average sleep, sleep inconsistency is an important indicator of sleep dysfunction. The current study sought to extend our knowledge of the relationship between sleep and inflammation through an examination of sleep inconsistency and inflammatory biomarkers.

**Methods:** Secondary analyses of the Survey of Midlife in the United States (MIDUS) sleep study were conducted. Five hundred thirty-three individuals completed nightly sleep diaries, actigraphy, and underwent a blood draw for the inflammatory biomarkers C-reactive protein, interleukin-6, and fibrinogen. Sleep inconsistency was derived from 7 consecutive nights of assessment and was operationalized as nightly fluctuations in the following variables: terminal wakefulness, number of awakenings, time in bed, sleep onset latency, and wake after sleep onset. Structural equation modeling was used to examine the influence of a latent average sleep and a latent sleep inconsistency variable on a latent inflammation variable. Models were subsequently adjusted for age, sex, BMI, health, and medication. Stratified models by sex were also analyzed.

**Results:** The average sleep model would not converge. The sleep inconsistency model fit the data well. A significant positive association between the latent factors sleep inconsistency and inflammation was observed (β = 10.18, *SE* = 4.40, *p* = 0.021), suggesting inconsistent sleep is associated with higher levels of inflammatory biomarkers. When stratified by sex, the association between the latent sleep inconsistency factor and inflammation was significant for women (β = 1.93, *SE* = 0.82, *p* = 0.018), but not men (β = 0.20, *SE* = 0.35, *p* = 0.566). The association between sleep inconsistency and inflammation weakened following multivariate adjustment (β = 6.23, *SE* = 3.71, *p* = 0.093).

**Conclusions:** Inconsistent sleep may be an associated feature of inflammatory dysfunction, especially in women. Future studies should build upon this preliminary work and examine these associations longitudinally and through treatment trials.

## Introduction

Sleep inconsistency, also called intraindividual variability and night-to-night variability in sleep patterns, has emerged as an important approach to quantifying sleep with implications for physical and mental functioning (1–3). Studies linking poor sleep to clinical health outcomes have traditionally focused on aggregate markers of sleep disturbance, such as average sleep duration and poor subjective sleep quality (2). For example, in one study all participants with self-reported short sleep (<5 h) and participants with self-reported long sleep (≥8.5 h) who also reported poor sleep quality had elevated allostatic load, measured by a multisystem biological risk index comprised of various biomarkers representing seven different physiological systems (4). Evidence suggests sleep inconsistency may provide unique information beyond that of average sleep (5). The importance of sleep inconsistency highlighted by previous studies mirrors a broader trend in medical research recognizing that the presence of individual-level variation in behavior and cognitive functioning may reflect impaired physiologic systems and brain processes (6–9). Likewise, sleep inconsistency may represent an underlying collection of dysfunction in biological systems involved in sleep-wake regulation, dysfunction that may be missed by only examining average sleep.

Biological mechanisms through which poor sleep adversely impacts health outcomes continue to be identified and include proinflammatory responses (10, 11), the sympathetic nervous system (12), the renin-angiotensin-aldosterone system (13), and endothelial renal functioning (14). Systemic inflammation has been one of the most intensely studied of these potential mechanisms (10). Markers of systemic inflammation, such as C-reactive protein (CRP), interleukin 6 (IL-6), and fibrinogen have been linked to poor sleep, with higher levels of markers associated with poorer sleep (15, 16). The association between sleep and inflammation may be most robust in women (17, 18). Systemic markers of inflammation have been also observed to be altered in several clinical samples with sleep disturbance including rheumatoid arthritis and end-stage renal disease (15, 19). While the relationship between sleep and inflammation is likely bidirectional, systemic inflammation may also mediate the association between sleep dysfunction and adverse clinical outcomes (20–22). However, several studies have failed to find an association between sleep characteristics and markers of inflammation (10, 23). One reason for these mixed findings may be the countervailing association between inflammation and sleep, as sleep dysfunction can alter inflammation and inflammation can also lead to sleep recuperation (15).

Due to the nuanced suspected associations between sleep and inflammation, aggregate measures of sleep, such as sleep duration and quality may not adequately capture the complex interrelationships that exist between sleep and inflammation. Sleep inconsistency, conversely, may serve as a more sensitive marker of these dynamic processes and as a more robust measure for studying the associations between sleep and inflammation (5). There are several reasons why sleep inconsistency and acute disruptions in an individual's circadian rhythm may drive inflammation. For instance, awakening someone from sleep typically results in acute inflammation (24); however, over a more prolonged period of awakening, there is a release of both proinflammatory and anti-inflammatory cytokines (25). Thus, while poor sleep quality overall is associated with a general inflammatory state (26), anti-inflammatory mechanisms following prolonged awakenings may represent a compensatory mechanism to disrupted circadian rhythms (25) and this balance may lead to greater inflammation overall. Additionally, the circadian clock is present in the majority of the body's cells and corresponds to a 24-h cycle (27), and inconsistent sleep may promote greater inflammation overall by continual disruption and resetting of these underlying cellular mechanisms. In fact, one study in older adults found that greater sleep inconsistency, defined as the within-person standard deviation of bedtime, waketime, and time in bed, was associated with greater circulating inflammatory markers IL-6 and Tumor Necrosis Factor-alpha (28). The current study aims to extend these previous findings by investigating the association between sleep inconsistency and systemic inflammation in middle-aged and older adults. We hypothesized that inconsistency in sleep would be associated with greater systemic inflammation.

## Materials and Methods

### Procedures

Data from the National Survey of Midlife Development in the United States (MIDUS) 2, the second wave of a longitudinal study of health and well-being, were used for all analyses. The MIDUS study involved numerous ancillary studies, including investigations focused on sleep and inflammation (29). Of the 1255 participants in the MIDUS 2 biomarker project, data from the 533 participants who had actigraphy data collected at the University of Wisconsin-Madison were included in analyses. Participants in the MIDUS study provided written informed consent, and study procedures were approved by the Education and Social/Behavioral Sciences and the Health Sciences Institutional Review Boards at the University of Wisconsin-Madison. In order to participate in the MIDUS 2 biomarker project, individuals had to have met eligibility criteria for and participated in MIDUS 1 (i.e., aged 25–75, English speaking, non-institutionalized, and living in the coterminous United States) and have completed the MIDUS 2 phone survey and self-administered questionnaire (30).

### Measures

#### Sleep

Sleep data were collected on participants for 7 consecutive days and nights. Participants were asked to wear a Mini-Mitter Actiwatch®-64 (Koninklijke Philips N.V., Amsterdam, Netherlands) on their non-dominant wrist, record bedtime and rise time using the event marker function of the watch, and complete a sleep diary daily. Both sleep diaries and event markers were used to determine bedtimes and rise times for each participant. Additionally, using Actiware 5 software and a medium threshold for sleep/wake detection, sleep onset latency (SOL: amount of time to fall asleep), number of awakenings (NWAK: count of nocturnal awakenings), wake after sleep onset (WASO: amount of time awake during the night), and terminal wakefulness (TWAK: amount of time awake in the morning prior to rising from bed) variables were generated (5). To assess average sleep and sleep inconsistency, the individual means and standard deviations of SOL, NWAK, WASO, and TWAK were calculated using actigraphy data. Additionally, the individual means and standard deviations of time in bed (TIB: total amount of time spent in bed at night) using data available from the sleep diaries were calculated. Participants had exceptional compliance to the sleep assessments, with a mean number of missing actigraph data points of 0.18 (SD = 0.65). The median number of missing sleep measurements was 0 (range 0–4).

#### Inflammation

Methods for specimen collection and processing are described elsewhere (31), but briefly, venous blood for measurement of CRP, IL-6, and fibrinogen was collected between 06:30 and 07:00 from the non-dominant arm, when possible, into vacutainer tubes. For assays requiring serum (i.e., IL-6, high-sensitivity CRP) red/black serum separator tube(s) were used, and blue citrated tubes were used for assays requiring plasma (i.e., CRP and fibrinogen). Concentrations of IL-6 in serum samples were determined by using high-sensitivity enzyme-linked immunosorbent assays (ELISA; Quantikine® High-sensitivity ELISA kit #HS600B, R & D Systems, Minneapolis, MN) with a standard curve, for an assay range of 0.156–10 pg/mL. Citrated plasma levels of CRP were determined by a particle enhanced immunonepholometric assay and BNII nephelometer (Dade Behring, Deerfield, IL) for an assay range of 0.175–1,100 ug/mL. CRP samples falling below the assay range were re-analyzed in sera utilizing immunoelectrochemiluminescence and a high-sensitivity assay kit (kit #K151STG, Meso Scale Diagnostics, Rockville, MD) for an assay range of 0.014–216 ug/mL. Fibrinogen was measured in citrated plasma samples using the BNII nephelometer (N Antiserum to Human Fibrinogen; Dade Behring, Deerfield, IL) for an assay range of 60–1,200 mg/dL.

These three cytokines were selected for investigation for several reasons. CRP is an acute-phase protein that increases in the body's response to inflammation. Higher levels of CRP are seen across a number of disease states including diabetes and heart disease (32). IL-6 is a critical pro-inflammatory cytokines and is involved in the up or down-regulation of the inflammatory cascade, guiding the body's immune response (33). Both IL-6 and CRP are more strongly related to psychosocial factors when compared to other cytokines (34). Finally, fibrinogen is a protein that aids in tissue repair and increases with greater inflammation, (35) and has been previously linked to poor sleep patterns (36).

#### Covariates

Potential demographic and clinical covariates included age (years since birth), sex (male/female), BMI (self-reported weight in kilograms/self-reported height in m^2^), health (0–10 self-rating of health from “worst possible health” to “best possible health,” respectively), and medication (sum of three yes/no variables: antihypertensive, antidepressant, and cholesterol-lowering medication).

### Data Analyses

Continuous covariates were summarized using means and standard deviations and categorical covariates using frequencies and percentages. Indicators of average sleep and sleep inconsistency were computed as the 7-days within-person mean sleep and standard deviation, respectively. Within-person variables were summarized using means and standard deviations. All analyses were performed using the lavaan package in R (37, 38), using a full-information maximum likelihood (FIML) technique which handles missing data within the analysis thereby using all available data (39). An alpha level of 0.05 was used for all tests, where appropriate.

#### Structural Equation Modeling

**Figure 1:**
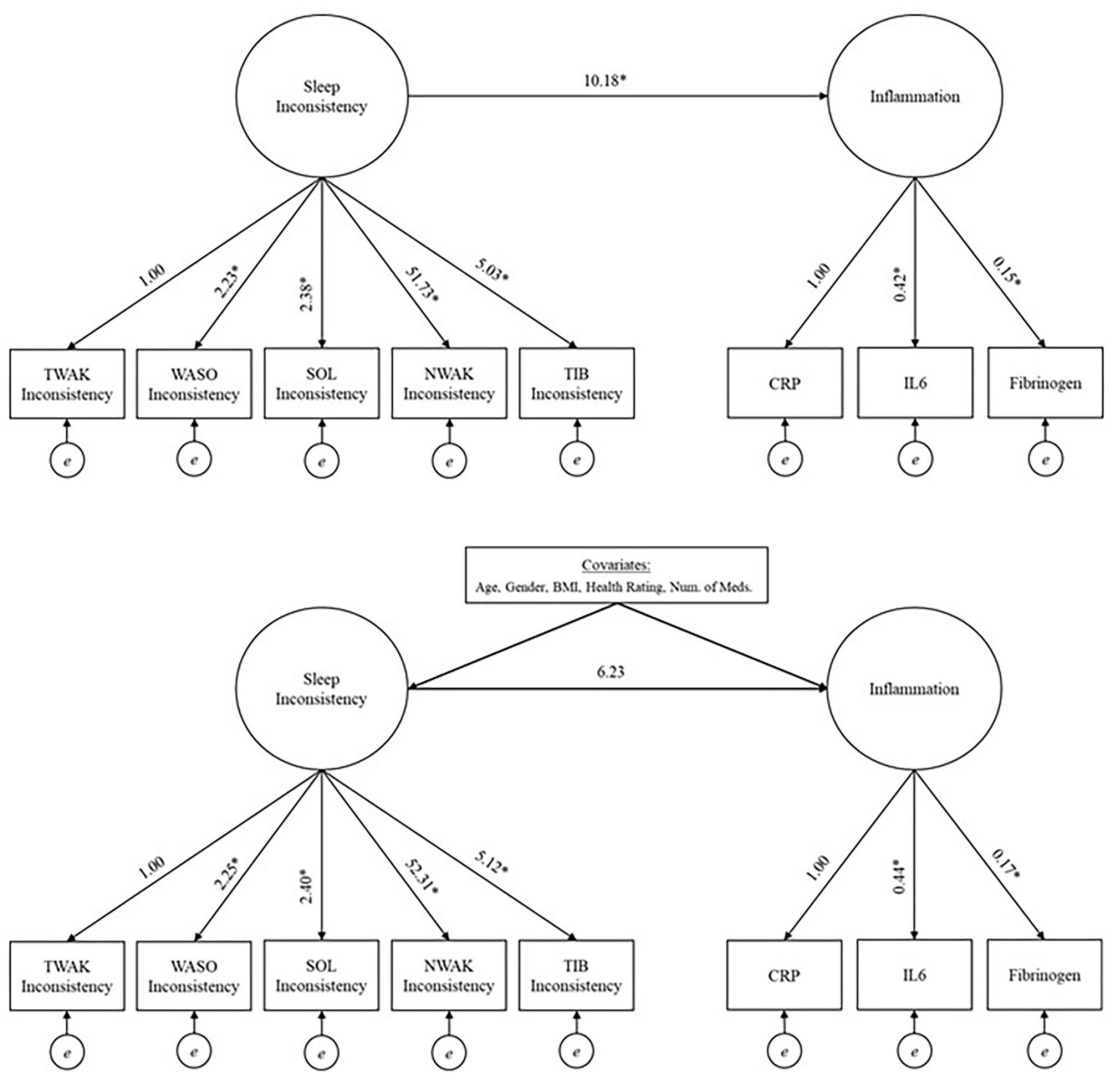
Unadjusted (top panel) and adjusted models (bottom panel) investigating the relationship between sleep inconsistency and inflammation. *Significant at 0.05 level. TIB, time in bed; NWAK, number of nightly awakenings; TWAK, terminal wakefulness; WASO, wake after sleep onset; SOL, sleep onset latency; CRP, C-reactive protein; IL-6, interleukin-6.

In order to examine the relationship between the latent factors, sleep and inflammation, structural equation modeling was used. A model with average sleep and a model with sleep inconsistency were built. Both models employed a two-step modeling approach to assess model fit (40). First, confirmatory factor analysis was used to validate and check the model fit of the measurement model, which included two-factors, either average sleep or sleep inconsistency and inflammation. The latent sleep factors were defined with either mean-values or within-person standard deviation values of TIB, NWAK, WASO, TWAK, and SOL; while a latent inflammation factor was defined with CRP, IL-6, and fibrinogen. SOL, TWAK and WASO inconsistency had variances several times larger than the other variables in the model so they were rescaled by a factor of 1/100 in the fitted model to place them on a similar scale to the other variables (rescaling has no impact on assessment of model fit). Once a good fit of the measurement model was established, the full theorized, unadjusted model was fit and a subsequent multivariate adjusted model (with predetermined covariates; Figure 1 for unadjusted and multivariate adjusted models) (40). Models were also examined stratified by sex to explore possible heterogeneity in the sleep inconsistency to inflammation association.

#### Fit Indices

Model fit indices were evaluated for the measurement model, unadjusted model, and multivariate adjusted model, and the normalized residual covariance was examined to evaluate local fit of the model in any cases where the global fit indices indicated poor fit. All models described were assessed for global and local model fit issues. The Comparative Fit Index (CFI), Root Mean Square Error of Approximation (RMSEA), Standardized Root Mean Square Residual (SRMR), and the Chi-Squared Test of Model Fit were used to assess global fit. A CFI >0.90 or >0.95, RMSEA or SRMR <0.05, or non-significant Chi-Square value were all indications of good model fit. In order to assess local fit, the standardized residual covariance matrix was evaluated, and residuals >2 were considered an indication of an area in which the model was not adequately fitting the data.

## Results

Descriptive statistics for the covariates and observed sleep inconsistency and inflammatory variables are provided in Table 1. The coefficients of variation for the inflammatory biomarkers are as follows: 1.61, 0.93, 0.25 for CRP, IL-6, and fibrinogen, respectively. The measurement model for average sleep failed to converge, and subsequent analyses were not performed. The fit indices for the second measurement model (i.e., model building latent sleep inconsistency and inflammation variables from observed sleep and inflammation indicators), including the CFI (0.978), RMSEA (0.040), and SRMR (0.044), suggest good model fit (Table 2). The chi-squared test is significant (*p* = 0.015), but this result is expected given its sensitivity to large sample sizes. Overall, the measurement model fit well and was used to examine the full structural model, both unadjusted and multivariate adjusted models.

**Table 1:** Descriptive statistics for covariates, sleep characteristics, and inflammatory markers.

	**Mean (SD) or Frequency (%)**
Gender	
Male	213 (39.96%)
Female	320 (60.04%)
Age	56.12 (11.56)
Race—White	314 (94.6%)
BMI	30.76 (7.23)
Self-rated health (0: Worst–10: Best)	7.60 (1.40)
Number of medications	2.23 (0.86)
Average sleep	
TIB	8.22 (2.55)
NWAK	32.47 (15.05)
TWAK	16.74 (15.05)
WASO	49.00 (36.40)
SOL	31.27 (49.22)
Sleep inconsistency	
TIB	1.22 (1.34)
NWAK	9.29 (5.31)
TWAK	22.18 (36.13)
WASO	20.32 (18.70)
SOL	26.60 (29.47)
Inflammatory markers	
CRP (ug/mL)	3.56 (5.74)
IL-6 (pg/mL)	3.44 (3.21)
Fibrinogen (mg/dL)	356.24 (89.09)

*TIB, time in bed measured in hours; NWAK, number of nightly awakenings; TWAK, terminal wakefulness measured in minutes; WASO, wake after sleep onset measured in minutes; SOL, sleep onset latency measured in minutes; CRP, C-reactive protein; IL-6, interleukin-6. Average sleep was calculated as the 7-days average of each sleep characteristic. Sleep inconsistency was calculated as the 7-days individual standard deviation for each participant*.

**Table 2:** Fit indices from structural equation modeling predicting inflammation from sleep inconsistency.

		**Unadjusted models**			
**Model fit indices**	**Measurement model**	**Full model**	**Male model**	**Female model**	**Adjusted model**
Comparative fit index (CFI)	0.978	0.978	0.999	0.940	0.901
Root mean square error of approximation (RMSEA)	0.040	0.028	0.000	0.071	0.055
Standardized root mean square residual (SRMR)	0.044	0.038	0.052	0.073	0.073
Chi-Squared test *p*-value	0.015	0.015	<0.001	<0.001	<0.001

*The following values indicate good fitting models: CFI > 0.90 or > 0.95; RSMEA < 0.05; SRMR < 0.08; Chi-Squared Test p-value > 0.05*.

The model fit indices for the unadjusted model (i.e., the structural model, without covariates but including a path from the latent sleep inconsistency variable to the latent inflammation variable) are presented in Table 2, and the model results are presented in Table 3 and Figure 1 (top panel). The model exhibited good fit as specified by a CFI (0.978), RMSEA (0.028), and SRMR (0.038). The chi-squared test is significant (*p* = 0.015), but, as above, this result is expected in large sample sizes. The manifest sleep inconsistency variables all significantly loaded on the latent sleep inconsistency factor (all *p*'s < 0.003), and all manifest inflammation variables significantly loaded on the latent inflammation factor (all *p*'s < 0.001). In the unadjusted model, there is a significant positive relationship between the latent sleep inconsistency factor and the latent inflammation factor (β = 10.18, *SE* = 4.4, *p* = 0.021) indicating that those with greater sleep inconsistency had higher levels of inflammation.

**Table 3:** Results from structural equation modeling predicting inflammation from sleep inconsistency.

**Measurement model**	**Unadjusted model**			**Adjusted model**		
	**Factor loadings**	**Standard error**	***P*-value**	**Factor loadings**	**Standard error**	***P*-value**
**SLEEP INCONSISTENCY**						
TWAK	1.00	–	–	1.00	–	–
WASO	2.23	0.58	<0.001^*^	2.25	0.59	<0.001^*^
SOL	2.38	0.64	<0.001^*^	2.40	0.65	<0.001^*^
NWAK	51.73	13.77	<0.001^*^	52.31	14.06	<0.001^*^
TIB	5.03	1.71	0.003^*^	5.12	1.74	0.003^*^
**INFLAMMATION**						
CRP	1.00	–	–	1.00	–	–
IL-6	0.42	0.05	<0.001^*^	0.44	0.05	<0.001^*^
Fibrinogen	0.15	0.02	<0.001^*^	0.17	0.02	<0.001^*^
**Regressions**	**Structural coefficients**	**Standard error**	***P*****-value**	**Structural coefficients**	**Standard error**	***P*****-value**
**INFLAMMATION**						
Sleep inconsistency	10.18	4.40	0.021^*^	6.23	3.71	0.093
Age	–	–	–	0.05	0.02	0.008^*^
Sex	–	–	–	1.31	0.38	0.001^*^
Medication	–	–	–	−0.37	0.24	0.132
BMI	–	–	–	0.18	0.03	<0.001^*^
Health	–	–	–	−0.60	0.19	0.002^*^

* *Significant at the 0.05 level. TIB, time in bed; NWAK, number of nightly awakenings; TWAK, terminal wakefulness; WASO, wake after sleep onset; SOL, sleep onset latency; CRP, C-Reactive Protein; IL-6, interleukin-6. Sleep inconsistency was calculated as the 7-days individual standard deviation for each participant. Unstandardized coefficients, as presented in this table, reflect the expected linear change in y (inflammation) for each unit increase in x (i.e., manifest variable or covariate)*.

The model fit indices for the unadjusted model stratified by sex are presented in Table 2, and the model results are presented in Table 4. The models exhibited good fit as specified by a CFI (0.999, 0.940), RMSEA (0.000, 0.071), and SRMR (0.052, 0.073), respectively for the male and female models. The chi-squared tests were significant (*p*'s = < 0.0001); however, as above, this result is expected in large sample sizes. In the male model, the manifest sleep inconsistency variables did not significantly load onto the latent sleep inconsistency factor (all *p*'s > 0.05); however, the manifest inflammation variables did significantly load on the latent inflammation factor (all *p*'s < 0.001). In the male model, there was not a significant association between the latent sleep inconsistency factor and the latent inflammation factor (β = 0.20, *SE* = 0.35, *p* = 0.566). In the female model, the manifest sleep inconsistency variables did significantly load on the latent sleep inconsistency factor (all *p*'s < 0.002), and the manifest inflammation variables did significantly load onto the latent inflammation factor (all *p*'s < 0.001). In the female model, there was a significant association between the latent sleep inconsistency factor and the latent inflammation factor (β = 1.93, *SE* = 0.82, *p* = 0.018), suggesting that women with higher levels of inconsistent sleep also exhibit higher levels of inflammation.

**Table 4:** Results from unadjusted structural equation modeling predicting inflammation from sleep inconsistency stratified by sex.

**Measurement model**	**Male model**			**Female model**		
	**Factor loadings**	**Standard error**	***P*-value**	**Factor loadings**	**Standard error**	***P*-value**
**SLEEP INCONSISTENCY**						
TWAK	1.00	–	–	1.00	–	–
WASO	0.10	0.12	0.84	0.35	0.10	<0.001^*^
SOL	0.11	0.13	0.41	0.36	0.10	0.001^*^
NWAK	2.66	3.21	0.41	7.45	2.18	0.001^*^
TIB	0.19	0.25	0.77	1.08	0.34	0.002^*^
**INFLAMMATION**						
CRP	1.00	–	–	1.00	–	–
IL-6	0.45	0.08	<0.001^*^	0.43	0.06	<0.001^*^
Fibrinogen	0.13	0.03	<0.001^*^	0.16	0.02	<0.001^*^
**Regressions**	**Structural coefficients**	**Standard error**	***P*****-value**	**Structural coefficients**	**Standard error**	***P*****-value**
**INFLAMMATION**						
Sleep inconsistency	0.20	0.35	0.57	1.93	0.82	0.018^*^

* *Significant at the 0.05 level. TIB, time in bed; NWAK, number of nightly awakenings; TWAK, terminal wakefulness; WASO, wake after sleep onset; SOL, sleep onset latency; CRP, C-Reactive Protein; IL-6, interleukin-6. Sleep inconsistency was calculated as the 7-days individual standard deviation for each participant. Unstandardized coefficients, as presented in this table, reflect the expected linear change in y (inflammation) for each unit increase in x (i.e., manifest variable or covariate)*.

The model fit indices for the multivariate adjusted model (i.e., full model including covariates) are presented in Table 2 and the results are presented in Table 3 and Figure 1 (bottom panel). The model fit for the multivariate adjusted model is ambiguous, but several fit indices indicate that it may be less than adequate with both RMSEA and SRMR >0.05 and a significant chi-squared test (*p* < 0.001). However, the CFI (0.901) meets the minimal standard for good model fit. The manifest sleep inconsistency variables all significantly loaded on the latent sleep inconsistency factor (all *p*'s < 0.003), and all manifest inflammation variables significantly loaded on the latent inflammation factor (all *p*'s < 0.001). Age, sex, BMI, and health status were significant covariates, suggesting that these demographic characteristics help explain some of the variation in inflammation and should therefore be adjusted for in future studies. The relationship between the latent factors of sleep inconsistency and inflammation is weakened when controlling for covariates (β = 6.23, *SE* = 3.71, *p* = 0.093). The multivariate adjusted model would not converge when stratified by sex.

## Discussion

The present study sought to investigate possible associations between an emerging, novel quantification of sleep patterns, sleep inconsistency, and markers of systemic inflammation. Commensurate with our hypothesis, we found that greater sleep inconsistency was associated with greater inflammation. This association was most robust in women. Importantly, the observed sleep and inflammation variables all loaded onto common factors suggesting that both sleep inconsistency and inflammation were highly correlated across various measures. These common factors were not observed in aggregate (i.e., mean) levels of sleep quality, the more commonly used markers of sleep dysfunction (i.e., a model aimed at building a latent mean-level sleep variable would not converge). As such, our analytic approach may signify underlying shared physiologic patterns of sleep inconsistency and inflammation. Past research on associations between sleep and inflammation has resulted in mixed findings, possibly due to evaluating aggregate measures of sleep rather than examining sleep inconsistency. However, preliminary research on sleep inconsistency has begun to identify patterns of associations between sleep inconsistency and a variety of inflammatory outcomes. As mentioned previously, one study of older adults found that inconsistency in sleep was associated with elevated peripheral inflammatory cytokines (28). Another study demonstrated that even one night of sleep disruption, as occurs frequently with on-call physicians, can impact an individual's immune modulation (41). Furthermore, in adolescents, sleep inconsistency measured across 1 week via actigraphy, was associated with higher levels of CRP (42). Nevertheless, the literature on sleep inconsistency and inflammation in adults remains limited, and the present study provides further preliminary insight into this association.

Finally, we found that, after controlling for covariates (i.e., age, sex, BMI, health rating, and medication), the association between the sleep inconsistency and inflammation latent factors was no longer significant. Since the adjusted model was no longer significant after the addition of covariates, it can be assumed that part of the relationship between sleep inconsistency and inflammation is due to demographic and clinical factors that account for differences in sleep inconsistency and inflammation. Previous research has shown that age (43), sex (44), BMI (45), self-rated health (46), and medication use [e.g., antihypertensives (47)], are associated with changes in sleep. These clinical and demographic factors have also been independently associated with inflammation [i.e., age (48), sex (49), BMI (50), self-rated health (51)], and medication use [e.g., antidepressants (52)]. Therefore, the findings of the present study are consistent with prior literature evaluating demographic and clinical predictors of sleep disturbance and inflammation.

There are a number of mechanistic pathways that may explain the relationship between sleep inconsistency and inflammation. For instance, when individuals are woken from sleep on just one occasion, an inflammatory cascade unfolds that can be measured in altered gene expression (53) and multiple awakenings and inconsistent sleep from night-to-night may further exacerbate the inflammatory response. Inflammation increases during stages 1 and 2 of sleep, as well as rapid eye movement (REM) sleep, while levels of inflammation during slow wave sleep are comparable to levels of awakening hours (54). Thus, returning to a level of homeostasis during slow wave sleep (lower levels of inflammation) may represent an integral physiological function of the various stages of sleep. However, if an individual wakes up at different times each night—or goes to bed at highly inconsistent times each evening—an inflammatory profile may be promoted, eventually making its way “downstream” to the physiologic markers, such as CRP and fibrinogen, measured in this study. Such inflammatory profiles may be particularly pronounced in populations known to have increased proportions of REM sleep to slow wave sleep, such as older adults or those suffering from depression (55–57).

Other factors could contribute to our findings. First, the overall levels of inflammation in this sample deserve further comment. For example, CRP is considered clinically relevant above the cutoff of 3 mg/L (58). One-third of the population has minor elevation in CRP, defined as levels >3 mg/L but <10 mg/L (58). This minor elevation has been associated with a variety of health outcomes, including those associated with tissue damage or environmental irritants (58). In our sample, mean CRP was 3.56 mg/L, indicating that our sample fell within this mildly elevated range. This could indicate that the association between sleep inconsistency and inflammation could be unique to individuals with at least mild systemic inflammation. Second, the current findings are noteworthy in light of other studies of sleep inconsistency. For instance, while our goal was to take the initial step toward identifying a common sleep inconsistency factor and examine associations with inflammation, previous work has identified that sleep inconsistency may be moderated by sex (28). In the current analyses, females indeed showed greater levels of inflammation. These sex differences and minor elevation in inflammation specific to our sample could be responsible for at least some of the associations of sleep inconsistency with inflammation.

The results of this study may help guide research on the mechanisms linking altered sleep processes and circadian misalignment to adverse health outcomes. Sleep and circadian processes are involved in the regulation of inflammatory cytokines, and experimental manipulation of both has been shown to increase blood concentrations of inflammatory cytokines (59–61). Likewise, inconsistent sleep may reflect a pattern of altered sleep and circadian processes that have implications on inflammatory regulation and health. For example, recent evidence suggests that sleep inconsistency confers risk for cardiometabolic disease (62–65). Given that inflammation plays a critical role in the pathogenesis of metabolic abnormalities (66), research is needed to explore whether inflammation is a mechanism through which sleep inconsistency and metabolic health are linked. Because sleep inconsistency is readily modifiable, this line of research may lead to sleep interventions aimed at preventing diseases that have been linked to altered sleep, including cardiovascular disease. Further, our finding of an association between sleep inconsistency and inflammation in women but not men (when stratified by sex) may be the result of gender differences in biological and socioeconomic factors. Future research is warranted.

There are a number of limitations warranting further comment in this study. First, peripheral markers of inflammation were only available at one time point. Such cross-sectional data limits our ability to quantify inconsistency in inflammatory processes over time, which has been suggested as a meaningful approach to linking inflammation to other health outcomes (6). Despite the current study being limited in only capturing sleep inconsistency, future research should target comparison of inconsistency across physiologic systems when possible (e.g., endocrine output coupled with behavioral activity throughout the day). Second, we were limited by the health behavior and comorbidity variables available in MIDUS. In order to extend the current findings, future research should implement collection of more robust time series of data within individuals. Examining longer epochs of data collection would allow researchers to identify antecedents of changes in sleep patterns or potential response to intervention, such as cognitive behavioral therapy for insomnia. Third, sources of sleep inconsistency (i.e., social jetlag, sleep disorders) were not readily discernable in the dataset and should be examined in future studies to identify potential differential effects on inflammation. Fourth, longitudinal investigations are needed to investigate whether the cross-sectional results hold over time. Lastly, the racial composition of the present sample prohibited important analyses based on race.

This study identified a significant relationship between sleep inconsistency and inflammation. Specifically, a latent factor of sleep inconsistency was found to be related to an overall inflammatory factor; however, this association was no longer significant after controlling for covariates. These novel findings extend prior work linking poor sleep patterns to physiologic dysfunction, further underscoring sleep inconsistency as a meaningful approach to quantifying sleep. Future research should extend measures of inconsistency to other physiologic systems as well as measuring how sleep inconsistency changes over time in conjunction with markers of inflammation.

## Data Availability Statement

The datasets analyzed for this study can be found in the Midlife in the United States study website: http://www.midus.wisc.edu.

## Ethics Statement

The studies involving human participants were reviewed and approved by Education, Social/Behavioral Sciences, and Health Institutional Review Boards at the University of Wisconsin-Madison. The patients/participants provided their written informed consent to participate in this study.

## Author Contributions

JD, DK, and TS contributed to the conception and design of the study. KB organized the database and performed statistical analyses. JD wrote the first draft of the manuscript. JD, ED, DK, TS, and KB wrote sections of the manuscript. All authors contributed to manuscript revision, read, and approved the submitted version.

## Conflict of Interest

The authors declare that the research was conducted in the absence of any commercial or financial relationships that could be construed as a potential conflict of interest.
